# A biocomplex to repair experimental critical size defects associated with photobiomodulation therapy

**DOI:** 10.1590/1678-9199-JVATITD-2021-0056

**Published:** 2022-02-14

**Authors:** Daniela Vieira Buchaim, Jesus Carlos Andreo, Karina Torres Pomini, Benedito Barraviera, Rui Seabra Ferreira, Marco Antonio Hungaro Duarte, Murilo Priori Alcalde, Carlos Henrique Bertoni Reis, Daniel de Bortoli Teixeira, Cleuber Rodrigo de Souza Bueno, Cláudia Rucco Penteado Detregiachi, Adriano Cressoni Araujo, Rogério Leone Buchaim

**Affiliations:** 1 Graduate Program in Structural and Functional Interactions in Rehabilitation, University of Marilia (UNIMAR), Marília, SP, Brazil.; 2 Medical School, University Center of Adamantina (UniFAI), Adamantina, SP, Brazil.; 3 Center for the Study of Venoms and Venomous Animals (CEVAP), São Paulo State University (UNESP), Botucatu, SP, Brazil.; 4 Department of Biological Sciences (Anatomy), Bauru School of Dentistry, University of São Paulo (USP), Bauru, SP, Brazil.; 5 Graduate Program in Tropical Diseases, Botucatu Medical School (FMB), São Paulo State University (UNESP), Botucatu, SP, Brazil.; 6 Graduate Program in Clinical Research, Center for the Study of Venoms and Venomous Animals (CEVAP), São Paulo State University (UNESP), Botucatu, SP, Brazil.; 7 Department of Dentistry, Endodontics and Dental Materials, Bauru School of Dentistry, University of São Paulo (USP), Bauru, SP, Brazil.; 8 Graduate Program in Applied Dental Sciences, Bauru School of Dentistry, University of São Paulo (USP), Bauru, SP, Brazil.; 9 Department of Health Science, Unisagrado University Center, Bauru, SP, Brazil.

**Keywords:** Bone regeneration, Biomaterials, Bone substitutes, Fibrin sealant, Low-level laser therapy, Photobiomodulation therapy

## Abstract

**Background:**

The association of scaffolds to repair extensive bone defects can contribute to their evolution and morphophysiological recomposition. The incorporation of particulate biomaterials into three-dimensional fibrin bioproducts together with photobiomodulation therapy (PBM) has potential and can improve regenerative medicine procedures. The objective of this experiment was to evaluate the effects of PBM therapy on critical size defects filled with xenogenic bone substitute associated with fibrin biopolymer.

**Methods:**

A critical defect of 8 mm was performed in 36 Wistar male adult rats that were divided into four groups. Groups BC and BC-PBM were defined as controls with defects filled by a clot (without or with PBM, respectively) and groups XS and XS-PBM that comprised those filled with biocomplex Bio-Oss^TM^ in association with fibrin biopolymer. PBM was applied immediately after the surgery and three times a week every other day, with the parameters: wavelength of 830 nm, energy density 6.2 J/cm^2^, output power 30 mW, beam area of 0.116 cm^2^, irradiance 0.258,62 W/cm^2^, energy/point 0.72 J, total energy 2.88 J. Fourteen and 42 days after the surgery, animals were euthanatized and subjected to microtomography, qualitative and quantitative histological analysis.

**Results:**

The BC-PBM and XS-PBM groups had a similar evolution in the tissue repair process, with a higher density of the volume of new formed bone in relation to the groups without PBM (*p* = 0.04086; *p* = 0.07093, respectively). Intense vascular proliferation and bone deposition around the biomaterial particles were observed in the animals of the groups in which biocomplex was applied (XS and XS-PBM).

**Conclusion:**

PBM therapy allowed an improvement in the formation of new bone, with a more organized deposition of collagen fibers in the defect area. Biocomplex favored the insertion and permanence of the particulate material in bone defects, creating a favorable microenvironment for accelerate repair process.

## Background

Most bone defects such as fractures have the capacity for spontaneous regeneration, which leads to treatment by conventional therapies. The repair process begins with the formation of the clot, accompanied by an inflammatory process with proliferation of granulation tissue. With the formation of a bone callus and organization of the new bone in lamellae, the process can progress to complete remodeling and total repair. However, in cases of large defects, fractures with loss of segment and resection of tumors, the use of grafts (autografts, allografts and xenografts) or bone substitutes may be indicated to contribute to healing [1,2]. More than two million grafting procedures are performed worldwide per year, the second most frequent tissue transplant after blood transfusion [3,4].

The autologous graft is still considered the gold standard, as it has the necessary properties for bone regeneration, in terms of combined osteoconduction, osteoinduction and osteogenesis [5,6]. However, its availability is limited and morbidity at the donor site demonstrates the need to develop alternative materials. Tissue engineering methods [7] are used to develop new bone substitutes that restore, improve or prevent the deterioration of compromised tissue function [8-11]. The development of biomaterials for grafts with biocompatibility, biodegradability and osteoconductibility properties, allow the proliferation of osteoblasts within an adequate three-dimensional structure (scaffolds), which provide ideal conditions for bone neoformation [12,13].

Bio-Oss^TM^ is a mineral bovine hydroxyapatite with chemical composition similar to natural mineral bone. It has satisfactory osteoconductive properties as it allows the neoformation of capillaries, perivascular tissue and migration of cells from the recipient bed through a three-dimensional structure. It does not induce a local or systemic immune response due to the complete elimination of bovine bone proteins carried out by a process of physical sterilization and by procedures [13-16]. Biomaterials in the form of small granules are commonly used in clinical practice. However, these granules are difficult to retain in the recipient bed, mainly due to the various conformations of defects and also due to bleeding at the site [17]. 

To solve this problem, biocomplexes can be used by associating the biomaterial with platelet-rich plasma (PRP) or fibrin (PRF), as well as fibrin scaffolds, to form a moldable material preventing the dispersion of the granules [18-22]. Engineered biocomplexes also can be made of mesenchymal-derived cells and a 3D structure as scaffold, for defect repair or transplantation purpose [23]. Fibrin sealants are three-dimensional biological matrices that provide support, fixation and cell growth, as well as the presence of various growth factors and angiogenesis, important properties that qualify them as candidates to integrate a biocomplex [24-26]. 

The heterologous fibrin sealant, also known as fibrin biopolymer, is a genuinely Brazilian product derived from animals, biocompatible, and of low production cost in addition to adhesive, sealant, hemostatic, scaffold, and drug delivery properties [27-33]. This new fibrin sealant was evaluated in phase I/II clinical trial and have showed be safe, non-immunogenic in addition to good preliminary efficacy [34,35]. 

In order to recover the compromised anatomy and function, together with grafts, complementary therapies can be used to reduce bone healing time and, eventually, reduce complications in the regenerative process. Among them, photobiomodulation (PBM) therapy, using low-level laser (LLLT), stands out due to its satisfactory effects on bone metabolism and repair, with great osteogenic potential, as it is a non-invasive and relatively inexpensive method [36,37].

There are few studies using fibrin as scaffold for biomaterials, especially with the heterologous biopolymer, in association with the PBM protocol used in this experiment. Therefore, the objective of this experimental protocol was to evaluate the effects of photobiomodulation on critical size defects filled with a biocomplex composed of xenogenic bone substitute associated with fibrin biopolymer.

## Methods

### Ethical aspects of research

The study was conducted according to the guidelines of the Declaration of Helsinki, and was approved by the Institutional Review Board of the Ethics Committee on Teaching and Research in Animals of the Bauru School of Dentistry, University of São Paulo (FOB-USP), Brazil, Protocol CEEPA n. 019/2016, dated October 21, 2016.

### Selection and maintenance of animals

Thirty-six male Wistar rats (*Rattus norvegicus*) were used, aged 90 days, body mass of approximately 390g, from the bioterium of the University of São Paulo (Ribeirão Preto, SP, Brazil) and kept in the bioterium of the Bauru School of Dentistry (University of São Paulo, Bauru, SP, Brazil).

The animals were kept in conventional cages containing 4 animals each, with feeders and drinking fountains *ad libitum* (Nuvilab^TM^ rat chow, Nuvital, Colombo, PR, Brazil), in an air-conditioned environment, air exhaustion, period light-dark 12/12 hours, temperature 22ºC ± 2ºC, humidity 60% ± 10, max noise 70 dB. 

Initially, the animals went through a supervised quarantine period to reach the desired age and weight, to verify possible changes in behavior and feed consumption. The inclusion criteria used were: males, healthy and young adults to avoid interference from hormonal factors, to ensure metabolic and physiological conditions.

### Randomization of experimental groups

The rats were randomly assigned to four groups according to the type of filling of the defect, and submitted to treatment by PBM: blood clot group (BC, *n* = 8), defect filled by blood clot obtained from the animal itself by cardiac puncture; blood clot with PBM group (BC-PBM, *n* = 8), defect filled by blood clot associated with PBM; xenogeneic bone graft + fibrin biopolymer scaffold group (XS, *n* = 10), defect filled by the association of the xenogenic biomaterial with the fibrin biopolymer; xenogeneic bone graft + fibrin biopolymer scaffold with PBM group (XS-PBM, *n* = 10), defect filled by the association of the xenogenic biomaterial with the fibrin biopolymer associated with PBM therapy.

## Scaffolds

### Xenogeneic bone graft

**Figure 1.**
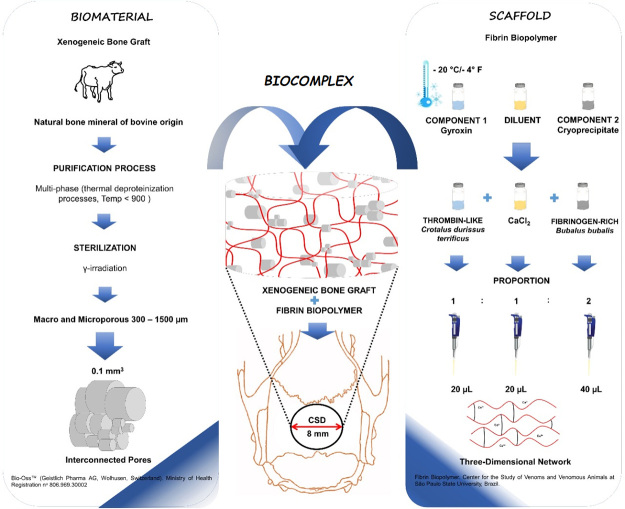
Schematic drawing of the production of the scaffolds used in this experimental protocol, the xenogeneic bone graft (Bio-Oss^TM^) and the fibrin biopolymer (CEVAP/UNESP). In the center of the image, the incorporation of the granular particles of the biomaterial into the fibrin biopolymer is represented, forming the biocomplex that was the filling material for bone defects of 8 mm in diameter in the center of the parietal bones of all animals.

The Bio-Oss^TM^ bone substitute (Geistlich Pharma AG, Wolhusen, Switzerland Wolhusen, Switzerland) that was used in this experimental protocol had granules of dimensions between 0.25-1 mm, packaged in a 2 g bottle, batch 81600891, ANVISA Registry nº 80696930002 (Figure 1).

### Heterologous fibrin biopolymer

Fibrin biopolymer was kindly provided by the Center for the Study of Venoms and Venomous Animals (CEVAP) at the São Paulo State University Júlio de Mesquita Filho (UNESP), Botucatu (São Paulo, Brazil), whose components and application formula are available at Ferreira Jr. et al. [38] and others [27,39].

The bioproduct consists of a thrombin-like enzyme purified from *Crotalus durissus terrificus* snake venom (component 1), the diluent comprises calcium chloride; and component 2 containing fibrinogen-rich cryoprecipitate extracted from *Bubalus bubalis* buffalo blood. All vials were stored in a freezer at ‒20°C until use [38] (Figure 1).

### Experimental surgery

Surgical procedures were performed at the Mesoscopy of the Anatomy Laboratory of the Department of Biological Sciences at FOB-USP. For surgery, the animals were submitted to general anestesia by intramuscular application of the combination of ketamine hydrochloride 50 mg/kg of animal weight (Dopalen^TM^, Seva Animal Health, Paulínia, SP, Brazil) and xylazine 10 mg/kg of animal weight (Anasedan^TM^, Seva Animal Health, Paulínia, SP, Brazil), in the proportion of 1:1.

Then, trichotomy was performed, with the aid of a hair trimmer (PhilipsT^M^ Multigroom QG3250, SP, Brazil), in the region of the frontal and parietal bones. In the animals of the BC and BC-PBM groups, cardiac puncture was performed by removing 1.5 to 2 mL of blood from each animal with a 5 mL syringe. The collected blood was transferred to a microtube to form the clot and be deposited in the bone defect. Antisepsis of the trichotomized region, including the coat around that area, was performed with a 10% topical solution of Polyvinyl Pyrrolidone Iodine PVPI (Povidine^TM^, Vic Pharma, Taquaritinga, SP, Brazil). The surgical procedure was always performed by the same surgeon.

The animals were fixed to the operating table, positioned in the prone position. Then, a 4 cm semi-lunar incision was made with a carbon steel scalpel blade No. 15 (Embramax^TM^, SP, Brazil) in the tegument and the periosteum was carefully detached with the aid of the syndesmotome and folded together with the other tissues, exposing the outer surface of the parietal bones. A circular osteotomy of 8.0 mm in external diameter was performed on the parietal bones, involving the sagittal suture, with the aid of the trephine drill (Neodent^TM^, Curitiba, PR, Brazil) adapted to the electric contra-angle (Driller^TM^, SP, Brazil) coupled to an electric micromotor (Driller^TM^, SP, Brazil), at low speed (1500 rpm), under constant and abundant sterile saline irrigation (0.9% saline) to prevent osteonecrosis by thermal action.

**Figure 2.**
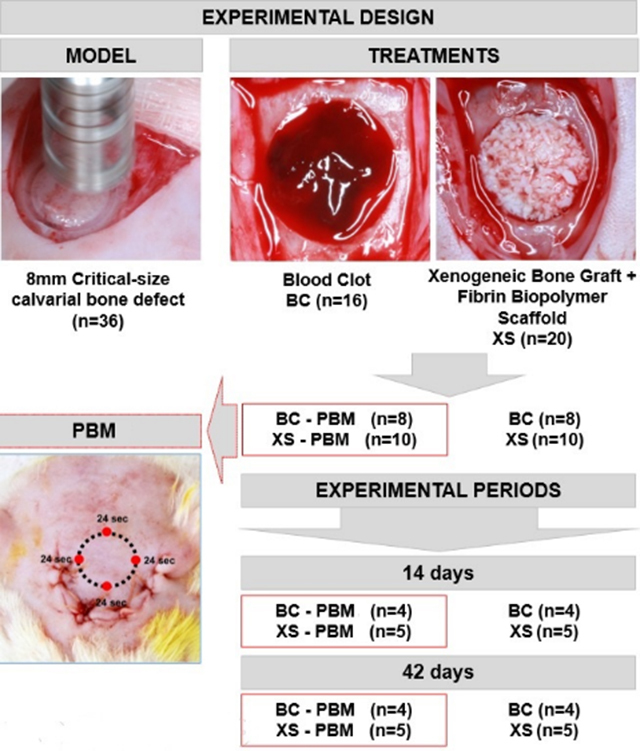
Experimental design. Thirty-six male Wistar rats underwent a critical bone defect in the center of the parietal bones with a no. 8 drill. In 16 animals, the defect was filled with a blood clot from an intracardiac puncture. In 20 animals, a biocomplex formed by the granulated biomaterial Bio-Oss^TM^ was incorporated into the fibrin biopolymer (CEVAP/UNESP). The groups were constituted as follows: blood clot group (BC, *n* = 8), defect filled with blood clot obtained from the animal itself by cardiac puncture; blood clot with PBM group (BC-PBM, *n* = 8), defect filled with blood clot associated with PBM; xenogeneic bone graft + fibrin biopolymer scaffold group (XS, *n* = 10), defect filled with the association of the xenogenic biomaterial with the fibrin biopolymer; xenogeneic bone graft + fibrin biopolymer scaffold with PBM group (XS-PBM, *n* = 10), defect filled with the association of the xenogenic biomaterial with the fibrin biopolymer associated with PBM. PBM was performed at four points in the form of a cross, for 24 seconds at each point, using the GaAlAs laser (Ibramed^TM^).

In all animals of groups XS and XS-PBM the defects were filled with fibrin biopolymer and Bio-Oss^TM^, with the biomaterial having a weight of approximately 0.03 mg. After complete polymerization of the biopolymer with the xenograft, the resulting biocomplex was transferred to the defect site without putting pressure on the brain. The proportion used (1:1:2 - 20 µL component 1, 20 µL diluent and 40 µL component 2) was in accordance with the recommendations of the producers and the amount readjusted according to the research needs (Figure 2).

The tissues of the surgical area were repositioned, taking care that the periosteum covered the cavity, and then the integument (simple stitches) was sutured with 5-0 silk thread (Ethicon^TM^, Johnson and Johnson Company, SP, Brazil). The region was carefully cleaned with gauze moistened with topical antiseptic, 2% chlorhexidine (Riohex^TM^, Rioquimica, São José do Rio Preto, SP, Brazil).

The animals were placed in the lateral decubitus position in cages, close to a light for adequate room temperature during all anesthetic recovery. Immediately after the surgical procedures, the animals received the analgesic paracetamol (Generic Medication, Medley^TM^, Suzano, SP, Brazil) at a dose of 200 mg/kg, 6 drops/animal dissolved in the water available in the drinking fountain.

Photobiomodulation (PBM) therapy

For PBM therapy, the animals were immobilized manually and carefully, making it unnecessary to use anesthetic during application. All animals in groups BC-PBM and XS-PBM were submitted to treatment with laser GaAlAs (gallium-aluminum-arsenide, Ibramed Laserpulse^TM^, Amparo, SP, Brazil). The PBM protocol, used for tissue repair in previous experiments [20,29,40-44], is described in Table 1.

**Table 1.** Details of the parameters used for PBM therapy.

Parameter	Description
Type of laser	Infrared (GaAlAs^*^)
Wavelength (nm)	830
Output power (mW)	30
Beam area (cm^2^)	0.116
Irradiance (W/cm^2^)	0.258,62
Treatment time per point of irradiation (s)	24
Number of irradiation points	4
Energy per point (Joule - J)	0.72
Total energy applied (Joule - J)	2.88
Energy density per point of irradiation (J/cm^2^)	6.20
Emission mode	Continuous
Application mode	Device held in contact mode and perpendicular to the skull
Frequency of treatment	3 times a week every other day

^*^Gallium-aluminum-arsenide.

The treatment started in the immediate postoperative period and three times a week every other day until the period corresponding to euthanasia (Figure 2). The laser beam emissions were calibrated on the device itself.

### Surgical procedure for tissue collection

After the periods of 14 and 42 days post-surgery, five animals from each group XS and XS-PBM and four animals from each group BC and BC-PBM, by period, were euthanized by the overdose method of general anesthetic (triple dose) used for surgery. Then, the defect region of each animal was removed, preserving the supraperiosteal soft tissues and fixed in a 10% formalin solution in pH 7.2 phosphate buffer for one week, and later, for examination on the microtomograph.

### Qualitative and quantitative analyzes

X-ray microtomography (micro-CT)

The collected pieces, after fixing with formalin, were submitted to an X-ray beam scan in the SkyScan 1174v2 computerized microtomograph (Bruker-microCT^TM^, Kontich, Belgium) of the Bauru School of Dentistry, Brazil. The X-ray beam sources (Cone-Beam) were operated at 50 kV, 800 uA, using a Cu + Al filter. The pieces were placed in tubes, positioned and fixed in the appropriate sample holder for the equipment, with wax pink, enabling stabilization and preventing movement during scanning. Then, they were rotated through 360º, with a rotation step of 0.5, and an isotropic resolution of 19.6 µm, generating an acquisition time of 41 minutes and 32 seconds per sample.

Each specimen images were analyzed and reconstituted using specific software 64Bits270013 (Bruker^TM^, Belgium) and the NRecon^TM^ Program (SkyScan, Bruker-micro CT) in about 1000 to 1100 slices according to the anatomical parameters adopted. The software Data Viewer^TM^ version 1.4.4 64 bit (linear measurements of the coronal, transaxial and sagittal axes) and CTvox^TM^ version 2.4.0 r868 (Bruker Micro CT), were used for two-dimensional visualization and then, the qualitative analysis of the tissue neoformed bone.

### Histotechnical processing

After microtomography, the pieces were washed in running water for 24 hours and subjected to demineralization in EDTA (4.13% tritiplex^TM^ III, Merck KGaA, Hessen, Germany and 0.44% sodium hydroxide^TM^, Labsynth, São Paulo, Brazil), with weekly changes of the solution for a period of approximately 6 weeks. 

After complete demineralization, they were dehydrated in an increasing series of ethyl alcohol, diaphanized in xylol and included in Histosec^TM^ processed paraffin (Merck, Hessen, Germany). Coronal slices were made, semi-series considering the central region of the defect with the aid of the semi-automatic microtome Leica^TM^ RM2245 (Leica Biosystems, Wetzlar, Germany) and 5 µm thick slices (6 slides with 4 cuts each) were obtained for hematoxylin-eosin (HE) staining and Picrosirius-red.

In the slides with picrosirius-red, the quality of the new bone formed in the defects was evaluated by structured collagen orientation. Thus, the images were obtained using the Leica DFC 310FX high resolution digital camera (Leica^TM^, Microsystems, Wetzlar, Germany) connected to the Leica DM IRBE confocal laser microscope and LAS 4.0.0 capture system (Leica^TM^, Microsystems, Heerbrugg, Switzerland ). Each type of fiber by color was evaluated using the analysis software of Axio Vision Rel. 4.8 Ink (Carl Zeiss^TM^ MicroImaging GmbH, Jena, Germany). The interlaced bone was recognized for its random and unorganized fibrillar pattern, usually with polarization colors varying between red-orange (immature bone tissue) and lamellar bone (light green/yellow), depending on the width of the fiber.

In the HE slides, the entire extension of the defect was considered to assess the bone repair pattern in all groups. Thus, it was possible to analyze in each defect the presence of granulation tissue, inflammatory infiltrate, the presence and quality of immature or mature/lamellar bone and the degree of filling of the newly formed tissue.

### Histomorphometric analysis

Four semi-serial sections of the surgical bed for each defect were evaluated using an Olympus^TM^ BX50 light microscope (Olympus, Tokyo, Japan) and the photographs were captured in 4x lenses with the attached digital camera (Olympus^TM^ DP 71, Tokyo, Japan) using the DP Controller 3.2.1.276 image capture software (Olympus^TM^, Tokyo, Japan) with image size specifications of 4080x3072 pixels and spot 30%.

The volume density (VVi) was defined as the volume fraction occupied by a given constituent (graft, inflammatory infiltrate, connective tissue, bone tissue and bone marrow) of the whole (defect with the graft + reaction tissue), and can be obtained in histological sections as area fraction ie VVi = AAi. Thus, the assessment of volume density followed the protocol described below. After capturing images covering the entire defect using the 4x lens, the reconstruction of the entire defect was performed in the Adobe Photoshop CS6 program. Then, the entire defect was evaluated in the image analysis program AxioVision, where the total area analyzed (A) and the area occupied by each constituent in the defect (Ai) were determined by the PIXEL unit of measurement. VVi of each type of structure was calculated by the relation: VVi = AAi = Ai / A.100 [45].

### Statistical analysis

The data obtained for the percentage of new bone formed were subjected to analysis of variance (ANOVA), in order to verify the existence of effects of the different groups tested in each evaluated period. The homogeneity of the variances and normality of the residues, necessary assumptions for the conduction of ANOVA, were tested, respectively, by the Shapiro-Wilk and Bartlett tests. Subsequently, the averages were compared using the Tukey test (*p* ≤ 0.05). The effect of the period evaluated in each tested group was compared by Student's t test (*p* ≤ 0.05). All analyzes were conducted using the R software (R Core Team, 2017).

## Results

### Microtomographic evaluation

Descriptive analysis of 2D images obtained for the different experimental groups

**Figure 3.**
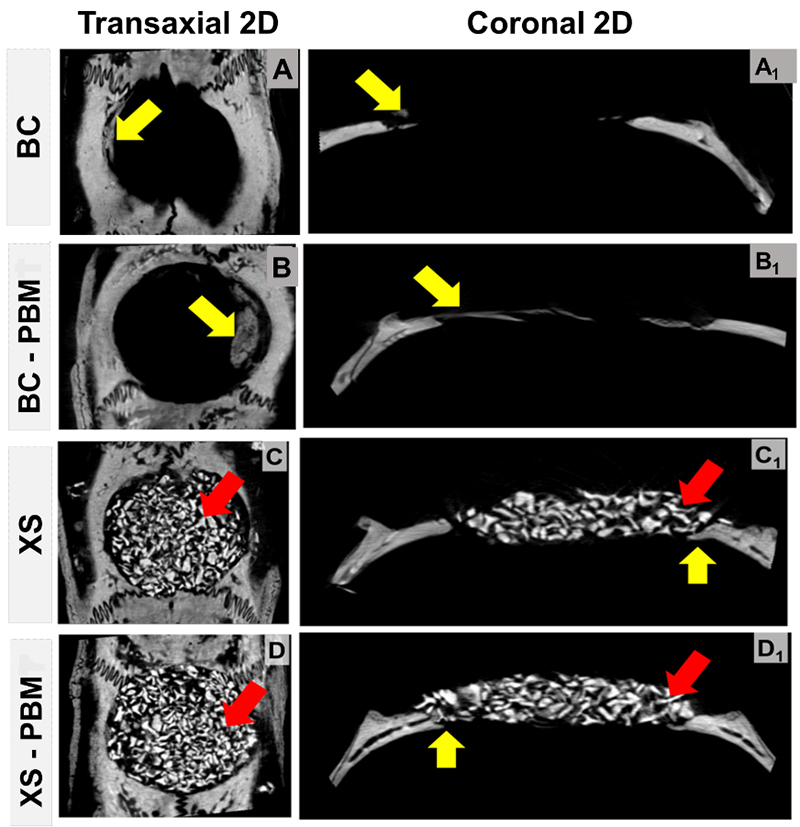
Two-dimensional microtomographic images, **(A-D)** transaxial and **(A1-D1)** coronal sections, which demonstrate the repair of critical defects in calvaria, treated with clot (BC and BC-PBM) and biocomplex composed of Bio-Oss^TM^ + fibrin biopolymer (XS and XS-PBM) within 14 days. Particles of the biomaterial (red arrow), thin trabeculae and newly formed bone tissue (yellow arrow).

**Figure 4.**
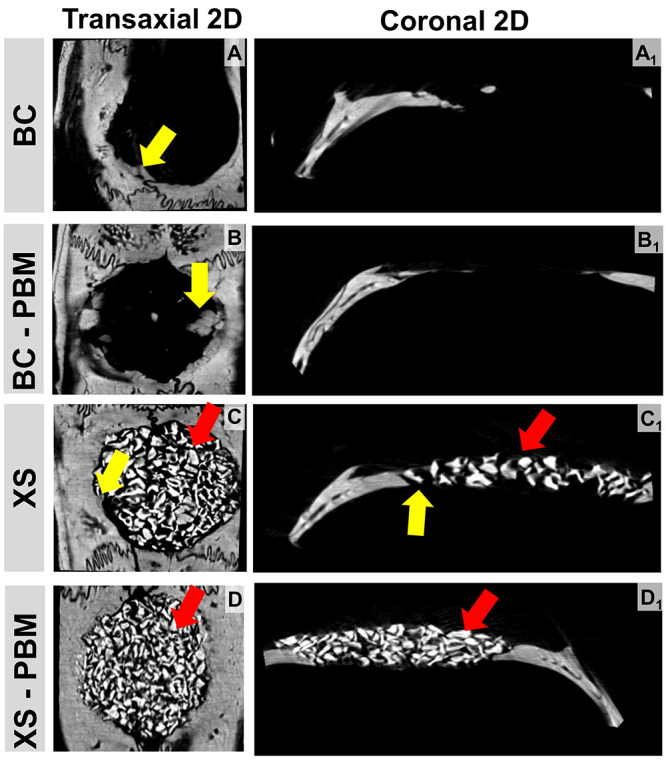
Two-dimensional microtomographic images, **(A-D)** transaxial and **(A1-D1)** coronal sections, which demonstrate the repair of critical defects in calvaria, treated with clot (BC and BC-PBM) and biocomplex composed of Bio-Oss^TM^ + fibrin biopolymer (XS and XS-PBM) within 42 days. Particles of the biomaterial (red arrow), thin trabeculae and newly formed bone tissue (yellow arrow).

The descriptive analysis of the microtomographic images in the periods of 14 and 42 days (Figures 3 and 4, respectively) was performed in the 2D plane (view of the transaxial and coronal sections), with the purpose of analyzing the evolution of the repair in the studied periods, the performance and maintenance of the biocomplex and, consequently, new bone formation.

In all evaluated defects, bone formation occurred centripetally, from the edges towards the center of the defect. In the BC and BC-PBM groups, although there was a continuous increase in bone neoformation during the experimental periods (Figures 3A-3B, 4A-4B and 3A1-3B1, 4A1-4B1), and in no animal did the complete defect close (yellow arrow) and the thickness of the bone formed was restricted to less than 1/3 of the original bone block surgically removed.

In the defect filled with the biocomplex (Figures 3C-3D, 4C-4D and 3C1-3D1, 4C1-4D1), the entire area of the defect was completely filled with Bio-Oss^TM^ particles, allowing the appearance of fine bone trabeculae from the edge of the defect and overlapping in the region of the dura mater within 14 days. A more expressive formation of bone tissue was observed in the group treated with laser photobiomodulation therapy XS-PBM compared to the XS group.

In the subsequent period (42 days) there was an increase in the amount of bone tissue, intertwining the biomaterial, in a more organized configuration. The Bio-Oss^TM^ particles were still very evident (red arrow). In some areas of the defect there was an absence of biomaterial and neoformed bone tissue in the XS-PBM group (yellow arrow). Some points of remodeled tissue were observed at the edges of the defect due to osteoclastic resorption.

Histomorphological observations

The repair occurred centripetally in all groups, with bone neoformation protruding from the margins to the center of the defect. It can be noted the absence of necrotic tissue, with the presence of bone cells and local neovascularization. There were no chronic inflammatory infiltrates with granuloma formation that characterized incompatibility of the grafted scaffolds with the recipient bed. Bone neoformation was partial, as indicated by the presence of areas without bone formation that were invaded by connective tissue. The neoformed bone matrix exhibited trabecular and mature characteristics, incorporating osteocytes derived from the differentiation of osteoblasts during the formation of the bone matrix.

### Morphological comparison between groups in the period of 14 days

*BC vs. BC-PBM*

**Figure 5.**
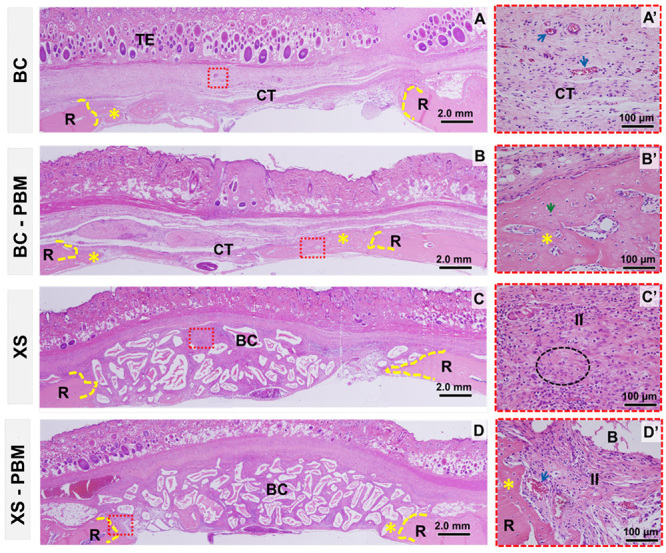
Histological images of critical size defects in rat calvaria within 14 days, coronal histological sections. **(A)** Blood clot group - BC; **(B)** blood clot with PBM group - BC-PBM; **(C)** xenogeneic bone graft + fibrin biopolymer scaffold group - XS; **(D)** xenogeneic bone graft + fibrin biopolymer scaffold with PBM group - XS-PBM. R: Remaining bone; TE: tegument; CT: connective tissue; BC: biocomplex biomaterial with fibrin biopolymer. B: Bio-Oss^TM^ particle; II: inflammatory infiltrate. Dotted yellow line: border; asterisk: bone neoformation; red dotted square: highlighted area; green arrow: osteocyte; blue arrow: blood vessel; black dotted circle: collagen fibers with scattered orientation. HE staining. **(A-D)** 4x lens, bar = 2 mm. **(A’-D’)** enlarged 40× images, bar = 100 µm.

All defects showed bone repair compromised in restoring height, due to the collapse of the integument into the defect, and in the conformation of the newly formed bone, which was irregular along the dura mater. In the BC group, the area of the defect was predominantly filled with loose connective tissue with small loci of bone neoformation on the edge of the defect, however in the BC-PBM the defects were partially filled with immature bone and visible greater vascular proliferation (Figure 5).

*XS vs. XS-PBM*

The defects were filled with a large amount of biomaterial of irregular size and conformation, surrounded by connective tissue. The reparative bone neoformation remained restricted to the edge of the defect, but more discreet in the XS group in relation to the XS-PBM. In the central region of the defect in both groups, it was observed in the loose connective tissue, particles of the biomaterial permeated by inflammatory cells and collagen fibers with different orientations (Figure 5).

### Morphological comparison between groups in the period of 42 days

*BC vs. BC-PBM*

**Figure 6.**
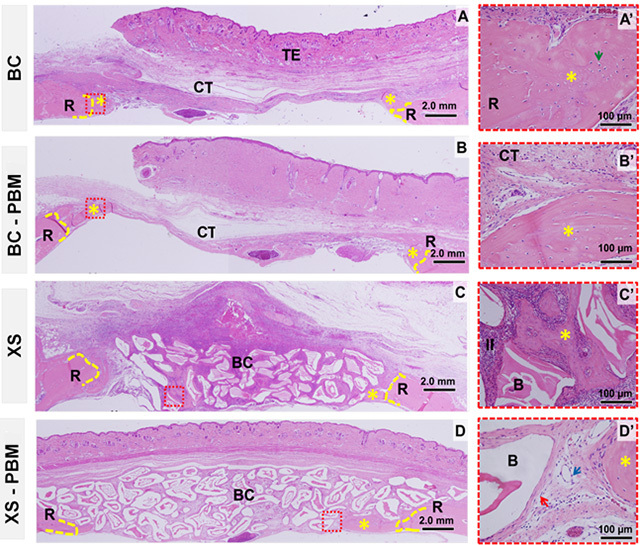
Histological images of critical size defects in rat calvaria within 42 days, coronal histological sections. **(A)** Blood clot group - BC; **(B)** blood clot with PBM group - BC-PBM; **(C)** xenogeneic bone graft + fibrin biopolymer scaffold group - XS; **(D)** xenogeneic bone graft + fibrin biopolymer scaffold with PBM group - XS-PBM. R: Remaining bone; TE: tegument; CT: connective tissue; BC: biocomplex biomaterial with fibrin biopolymer; B: Bio-Oss^TM^ particle; II: inflammatory infiltrate. Dotted yellow line: border; asterisk: bone neoformation; red dotted square: highlighted area; green arrow: osteocyte; blue arrow: blood vessel; red arrow: concentric collagen fibers. HE staining. **(A-D)** 4x lens, bar = 2 mm. **(A’-D’)** enlarged 40× images, bar = 100 µm.

At 42 days, in the BC group, the connective tissue formed filled the entire length of the defect, maintaining a thickness below that of the remaining bone and with less vascular formation, predominating in the region close to the bony edges. In the BC-PBM group, the defect was still filled with a large amount of connective tissue, showing a thin layer of bone tissue (asterisk) with diploid characteristics, and in some cases, they led to partial closure of the defect, but without recovery of your height (Figure 6).

*XS vs. XS-PBM*

In the panoramic aspect of the calvaria, an integrity of the defect height was observed due to the presence of the biomaterial and irregular border contour, resulting from the process of resorption of the graft particles, together with bone remodeling. In the XS group, there were also foci of inflammatory infiltrate mainly in the central area of ​​the defect. However, in the XS-PBM group, the presence of inflammatory infiltrate was diffuse in the interstitium and less intense.

During this period, bone neoformation was also restricted to the defect margins, but more evident than at 14 days. There was a gradual reabsorption of the biomaterial and an increase in bone tissue at the edges of the defect and on the surface of the particles located in the most central areas of the defect in the XS-PBM group. In most of the slides analyzed, thin concentric layers of collagen fibers were formed around the graft particles, with a more organized direction in the XS-PBM group (Figure 6). 

### Histomorphometry of the newly formed bone

**Figure 7.**
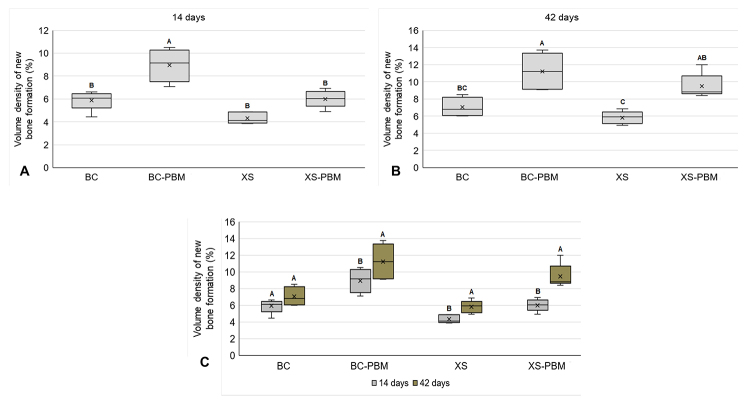
Volume density of new bone formed (%) in the different groups tested (BC, BC-PBM, XS, XS-PBM) in the periods of **(A)** 14 and **(B)** 42 days; ANOVA followed by Tukey's post-test (*p* ≤ 0.05). **(C)** Volume density of new bone formed (%) in each group tested in the evaluated periods (14 and 42 days); Student's t test (*p* ≤ 0.05). Different letters (A ≠ B ≠ C) indicate a significant difference (*p* ≤ 0.05).

At 14 days, there was a higher percentage of new bone formed in the XS-PBM group compared to the other groups, the difference being statistically significant (Figure 7A and Table 2). In the period of 42 days, the groups biostimulated with laser (BC-PBM and XS-PBM) had the highest averages, but with no significant difference between them. However, they showed a statistically significant difference in relation to their respective groups without photobiomodulation (BC and XS). There was no significant difference between XS-PBM and BC (Figure 7B and Table 2).

When bone formation was evaluated within the same group comparatively in the two experimental periods, a significant difference was observed between 14 and 42 days in all groups except BC. However, in 42 days, all had the highest averages of new bone formed (Figure 7C and Table 2).

**Table 2.** Percentage of new bone formation in each group in the two experimental periods (14 and 42 days).

	14 Days	42 Days	***p* Value**
BC	5.89 ± 0.85bA	7.06 ± 1.10bcA	0.1256
BC-PBM	8.93 ± 1.42aB	11.22 ± 2.10aA	0.04086
XS	4.31 ± 0.49bB	5.82 ± 0.73cA	0.02254
XS-PBM	6.01 ± 1.42bB	9.47 ± 1.45abA	0.01093

BC: blood clot group; BC-PBM: blood clot with PBM group; XS: xenogeneic bone graft + fibrin biopolymer scaffold group; XS-PBM: xenogeneic bone graft + fibrin biopolymer scaffold with pbm group.

Different lowercase letters (column, a ≠ b ≠ c) indicate a significant difference in the same period between groups (ANOVA and Tukey). Different capital letters (line, A ≠ B) indicate a significant difference in each group in the two periods (unpaired t-test). Values defined as the mean ± standard deviation (*p* ≤ 0.05).

## Discussion

This experimental protocol aimed to evaluate the repair of critical bone defects in rat calvaria using the association of two scaffolds forming a biocomplex - a xenogenic biomaterial widely used in regenerative medicine (Bio-Oss^TM^) and the heterologous fibrin biopolymer - and the associated photobiomodulation (PBM) therapy with the use of low-level laser (LLLT). The use of PBM enabled greater bone formation in the defect filled by a clot and also with the biocomplex, highlighting that the biocomplex creates a favorable microenvironment for an adequate repair process, especially in defects that do not repair spontaneously.

Bone defects can occur due to various clinical situations and their reconstruction is a necessary and important step to reestablish the morphological, functional and mechanical integrity of the compromised bone, aiming at the patient's rehabilitation [46]. Extensive bone injuries may have their potential for spontaneous regeneration compromised and leads to the search for the development of new osteosubstitute materials and three-dimensional bioadegradable scaffolds [1,47-49]. One of the most used experimental models in this area is the realization of critical defects in calvaria [50,51]. Critical defects can evolve to non-atrophic union due to the nature of the fracture, with impaired vascularity and soft tissue injury and will always require treatment of the defect [52].

In this experiment, it was observed in all groups, through transaxial and coronal microtomographic analysis, that the repair process with the formation of new bone, with radiopaque aspect in the images, occurred centripetally, from the margins of the surgical wound and the dura mater underlying. The evaluation was carried out qualitatively, since it becomes difficult to quantify with the presence of the graft, due to the similarity of radiopacity between the biomaterial and the new bone formed [53-55]. In addition, in this study, we chose to quantify new bone formed on histological slides, which allows greater precision in morphometry, avoiding conflicting and inadequate data [56,57].

During the post-surgical period of this experimental protocol, the defect treated by blood clot obtained from the animal itself through intracardiac puncture (groups BC and BC-PBM) did not completely close, being filled only by a thin bone layer on the surface of the dura mater, occupying 7.06 ± 1.10 and 11.22 ± 2.10 percent of the volume of the defects, respectively, at 42 days. Thus, the defect of 8 mm in diameter in the calvaria of rats was not able to repair spontaneously with only a blood clot and can be considered as a critical defect, in agreement with other similar experiments [55,58,59]. 

Histomorphometrically, at 14 days, the BC-PBM group showed the highest percentage of new bone formation (8.93 ± 1.42) with a significant difference in relation to the BC, XS and XS-PBM groups (5.89 ± 0.85, 4.31 ± 0.49 e 6.01 ± 1.42, respectively). All material grafted into a surgical cavity, even though it is biocompatible, generates an inflammatory response at the host site [9,60]. Biomaterials, after their implantation, delay bone deposition around the particles, being directly proportional to the reabsorption [61,62]. In addition, the PBM effects in the first days of its application, such as reducing the inflammatory response, can contribute to accelerating bone formation [37,63-66].

In the 42-day postoperative period, the BC-PBM and XS-PBM groups had a higher percentage of new bone deposition, with a significant difference in relation to the groups with the same treatment (11.22 ± 2.10 and 9.47 ± 1.45, respectively) and without PBM (BC 7.06 ± 1.10 and XS 5.82 ± 0.73, respectively). This fact occurs, among several biomodulatory benefits of LLLT, through the improvement in the lamellar organization of collagen fibers, increased bone cellularity, decreased inflammatory infiltrate and, thus, generate conditions for higher averages of neoformed bone [29,42,67-70].

When comparing the evolution of the repair process, within the same group, between the periods of 14 and 42 days, only the BC group did not show a significant difference (5.89 ± 0.85 and 7.06 ± 1.10, respectively). In general, due to the rapid reabsorption of the blood clot in the first days, there is the possibility of collapse/invasion of adjacent soft tissues that started to occupy the space of surgical defects. The insertion of the xenogenic biomaterial, mainly with the association of another three-dimensional scaffold such as the fibrin biopolymer, creates a structured microenvironment, mainly in the central area of the defects, which prevents the invasion of soft tissues and may even reduce the need for guided bone regeneration procedures, with the addition of membranes to cover the defect [20,29,71-74]

The surgical cavities in which the fibrin biopolymer (XS and XS-PBM groups) were used showed intense angiogenesis as early as 14 days, as well as the occasional presence of granulation tissue and the permanence of vascular spaces at 42 days [75]. This biological response demonstrates that the biopolymer is biocompatible, in agreement with previous studies [31,76,77], because in addition to not having a foreign body reaction, it favored the insertion in the surgical bed, the deposition and proliferation of osteoblastic cells, functioning as a scaffold for bone regeneration [78-82].

It can also be highlighted the binding function of the fibrin biopolymer, since in its use with the Bio-Oss^TM^ particles, it allowed the permanence in the implantation site forming multiple layers. As the biomaterials particulate in granules can be easily displaced from the surgical bed, even by the action of the local bleeding itself, it allows the use of these agents to be inducted with the purpose of forming a firm and mechanically stable network, with good adhesive properties [22,26,82]. The clinical applicability and benefits of commercially available fibrin sealants, derived from human blood, for soft tissues are well documented, but their contribution to bone surgery and oral and periodontal surgery remains controversial [83].

Finally, snake venom purified fibrin biopolymer is the only totally heterologous sealant in the world, with several beneficial properties in multiple areas of regenerative medicine. It has a low production cost, has proven to be clinically safe and effective in the treatment of chronic venous ulcers, and, through pre-clinical research carried out, a great potential for clinical applications such as scaffolding and drug delivery systems is foreseen [27,34,38,82,84-89]. The idea of using a biocomplex associated with PBM therapy by our research group opens a new opportunity to develop an innovative therapy to treat critical-size defects.

## Conclusion

In view of the results obtained, it can be seen that the defects treated with PBM therapy using an 830 nm wavelength laser allowed an improvement in the formation of new bone. This suggests that there was a biomodulation of the inflammatory process, with more organized deposition of collagen fibers in the defect area and consequently, a more homogeneous bone conformation. 

Therefore, the filling with the biocomplex formed by the association of the fibrin biopolymer and xenogeneic graft favored the insertion and permanence of the particulate material in the critical size defect performed in the calvaria of rats, creating a favorable microenvironment for an adequate repair process as an innovative drug delivery system.
